# Applying deep matching networks to Chinese medical question answering: a study and a dataset

**DOI:** 10.1186/s12911-019-0761-8

**Published:** 2019-04-09

**Authors:** Junqing He, Mingming Fu, Manshu Tu

**Affiliations:** 1 Key Laboratory of Speech Acoustics and Content Understanding, Institute of Acoustics, Chinese Academy of Sciences, Beijing, 100190 China; 2 University of Chinese Academy of Sciences, Beijing, 100049 China

**Keywords:** Medical question answering, Chinese word segmentation, Semantic matching, Convolutional neural networks, Deep learning

## Abstract

**Background:**

Medical and clinical question answering (QA) is highly concerned by researchers recently. Though there are remarkable advances in this field, the development in Chinese medical domain is relatively backward. It can be attributed to the difficulty of Chinese text processing and the lack of large-scale datasets. To bridge the gap, this paper introduces a Chinese medical QA dataset and proposes effective methods for the task.

**Methods:**

We first construct a large scale Chinese medical QA dataset. Then we leverage deep matching neural networks to capture semantic interaction between words in questions and answers. Considering that Chinese Word Segmentation (CWS) tools may fail to identify clinical terms, we design a module to merge the word segments and produce a new representation. It learns the common compositions of words or segments by using convolutional kernels and selects the strongest signals by windowed pooling.

**Results:**

The best performer among popular CWS tools on our dataset is found. In our experiments, deep matching models substantially outperform existing methods. Results also show that our proposed semantic clustered representation module improves the performance of models by up to 5.5% Precision at 1 and 4.9% Mean Average Precision.

**Conclusions:**

In this paper, we introduce a large scale Chinese medical QA dataset and cast the task into a semantic matching problem. We also compare different CWS tools and input units. Among the two state-of-the-art deep matching neural networks, MatchPyramid performs better. Results also show the effectiveness of the proposed semantic clustered representation module.

## Background

Automatic medical question answering is a special kind of question answering (QA) that is involved with medical or clinical knowledge. There is an urgent need to develop advanced automatic medical QA systems because of insufficient professionals and inconvenient access to hospitals for some people. According to an American health survey, 59% of U.S. adults had looked on the Internet for health information, among which 77% of them utilized the general search engines [1]. However, they have to filter numerous results of their queries to find desired information. For this sake, health consultancy websites have arisen, with thousands of medical professionals and enthusiastic patients answering the questions proposed by users. But this kind of service fails to provide immediate and accurate answers for users, which is unbearable for some patients. Moreover, medical QA systems also benefit physicians by providing previous answers from fellows as a reference.

### Tradictional Medical QA

The previous study on medical QA mainly focused on extracting answers from passages in books, health care records, and other clinical materials to assist in decision making [2]. Until now, remarkable progress has been made by researches and advanced information retrieval techniques have been applied to this task [3–6]. But these works were within a dominant paradigm of Evidenced-Based Medicine (EBM) that provides scientific evidence instead of a precise answer and only targeted at certain types of questions. These limitations made them inquisitive for patients and non-professional people.

Then on-line medical QA has been drawing the attention of scholars for its tremendous need. Jain and Dodiya presented rule-based architectures for online medical QA and introduced question processing and answers retrieval in detail [7]. However, rules failed to cover linguistic variety in practice. Wang et al. proposed to train word embeddings [8, 9] as semantic representation and evaluate the similarity between words as the correlation score between sentences [10]. However, all the methods above rely on well-designed templates, sophisticated features, and various manual tuning.

### Chinese Medical QA

Compared to English medical QA system, the research of Chinese QA in the medical field are immature and are still in a preliminary stage of development [2]. It is a challenging task that has two main difficulties:

1. Chinese word segmentation (CWS) performs worse in the medical domain than in open-domain. For dictionary-based methods, there are not publicly available Chinese clinical knowledge base and a standard of clinical terms like Systematized Nomenclature of Medicine (SNOMED). For data-driven methods, there are no annotated Chinese medical texts data to train a CWS tool. Moreover, there are unprofessional descriptions, typing errors, and abbreviations in on-line QA data. These phenomena also degrade the performance of CWS tools.
2. There are not enough Chinese medical QA datasets for study. Though there are data from challenges for promoting research on medical QA, including BioASQ challenges [11], CLEF tasks, and TREC medical tracks [12], none of them were in Chinese. To bridge the gap, we construct a large Chinese medical non-factoid QA dataset formulated in natural language, namely webMedQA, and make it publicly available.

Even so, Li combined multi-label classification scores and BM25 [13] values for question retrieval over a corpus of pre-built question-answer pairs [14]. He also applied the TextRank [15] algorithm to the re-ranking of candidates. His data were crawled from the web and not publicly available. The method was based on words and suffered from Chinese word segmentation failure in some cases. Then Zhang et al. proposed a multi-scale convolutional neural network (CNN, [16]) for Chinese medical QA and released a dataset [17]. (It is the only one that is publicly available as we know). This end-to-end approach eliminates human efforts and prevents from CWS failure by using character-based input. However, it uses the cosine distance as the similarity between the CNN representation of questions and answers, which could not capture the relation of words between questions and answers.

### Deep Matching in Open-domain QA

As for QA in open-domain, researchers have displayed meaningful work to select answers by semantic matching in various level. Hu et al. propose ARC-I and ARC-II, which first conducted word-level matching between sentences then applied CNNs to extract high-level signals from matching results [18]. Qiu and Huang then upgraded the structure of ARC-I by a tensor layer [19]. Later, Long-short term memory (LSTM, [20]) was adopted to construct sentence representations and used cosine similarity as scores [21]. Wan et al. further improved the representation by strengthening the position information using a bidirectional LSTM [22] and replaced the cosine similarity with multiple layer perceptron (MLP). Pang et al. then proposed MatchPyramid to extract hierarchical signals from words, phrase and sentence level using CNNs [23], which could capture rich matching patterns and identify salient signals such as n-gram and n-term matchings.

In this paper, we cast the QA task into a semantic matching problem that selects the most related answer. We first find the best CWS tools and the most suitable input unit for the task. Then we apply different state-of-the-art matching models in our task and compare them with baselines. We further propose a CNN-based semantic clustered representation (CSCR) to merge the word segments that are probably split wrong by CWS and produce a new representation that is compatible with deep matching models.

The main contributions of this work can be summarized as follows:

- We construct a large-scale comprehensive Chinese medical QA corpus for research and practical application. To our knowledge, it is the largest publicly available Chinese medical QA corpus so far.
- We propose a neural network to workaround the CWS problem for Chinese medical texts. It can semantically cluster characters or word segments into words and clinical terms then produce a word level representation. To the best of our knowledge, it is the first model to improve results of CWS inputs by post-processing.
- We apply semantic matching approaches to Chinese medical QA and conduct a serial of experiments on different input units and matching models. We build a brand new Chinese medical QA system using the best performer and report a benchmark result on our dataset.

## Methods

### Dataset Construction and Content

Our Chinese medical question answering (QA) data are collected from professional health-related consultancy websites such as Baidu Doctor [24] and 120Ask [25]. Users first fill in the form of personal information, then describe their sicknesses and health questions. These questions are open to all the registered clinicians and users until the question proposer choose the most satisfying answer and close the question. Doctors and enthusiastic users can provide their diagnoses and advice under the posted questions with their titles and specialize being displayed together with their answers. The questioners can also inquire further if they are interested in one of the answers, which is a rare case in the collected data. The category each question belongs to is also selected by its proposer.

We filtered the questions that have adopted answers among all the collected data, which add up to a total of 65941 pieces. Then we cleaned up all the web tags, links, and garbled bytes leaving only digits, punctuations, Chinese and English characters using our preprocessing tool. We also dropped the questions that their best answers are longer than 500 characters. The questions that have more than one best-adopted replies are also removed. Finally, we got a set of 63284 questions. We further sampled 4 negative answers for each question for related research such as answer ranking and recommendation. For the questions that have less than 4 negative replies, we randomly sampled answers from other questions for supplementation. Then we split the dataset into training, development and test sets according to the proportion of 8:1:1 in each category. Zhang et al. also introduced a Chinese Medical QA dataset (cMedQA) [17]. Comparison of these two open datasets is listed in Table 1. The statistics of the questions and answers in the training, validation and test sets are listed in Table 2. The average length of questions is shorter than the answers. All the lengths are similar between the training, development and test sets.

**Table 1:** Comparison of cMedQA and our webMedQA dataset

Dataset		cMedQA	webMedQA
# Ans	Train	94134	253050
	Dev	3774	31685
	Test	3835	31685
	Total	101743	316420
# Ques	Train	50000	50610
	Dev	2000	6337
	Test	2000	6337
	Total	54000	63284
Contain category		No	Yes

**Table 2:** The statistics of answers and questions in webMedQA dataset

	Train	Dev	Test
Number of Ans.	253050	31685	31685
Avg. Length of Ans.	146.88	147.74	148.50
Max Length of Ans.	500	499	499
Min Length of Ans.	2	2	2
Number of Ques.	50610	6337	6337
Avg. Length of Ques.	86.68	87.43	86.08
Max Length of Ques.	1312	1302	1150
Min Length of Ques.	2	3	5

**Fig. 1:**
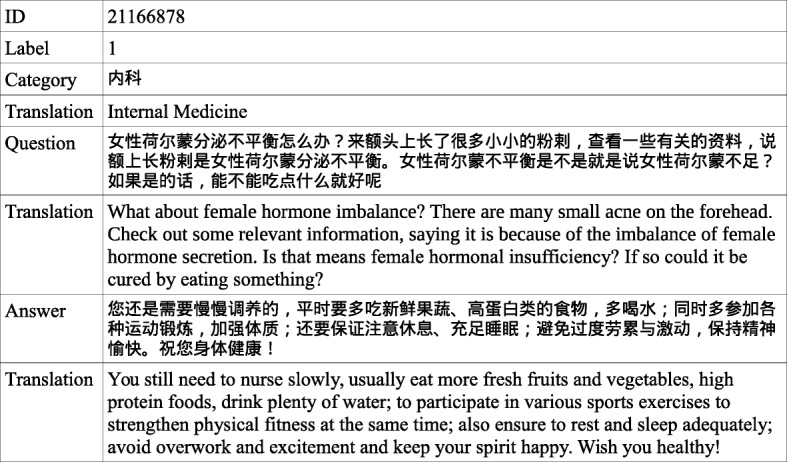
A sample in the webMedQA. The 5 fields are on the left with their contents on the right

In the webMedQA dataset, each line is a QA sample containing 5 fields: a question ID, a binary label of whether the answer is adopted, its category, the question, and an answer. They are all split by a tab. The ID is unique for each question and label 1 indicates the answer is correct. A clinical category is given for each sample but may be wrong in some cases. The translation of the clinical category, question and answer are listed in the cell under the original texts, which are not included in the dataset. A sample is given in Fig. 1.

There are 23 categories of consultancy in our dataset, covering most of the clinical departments of common diseases and health problems. The amount of the questions in each category in webMedQA dataset are listed in Table 3. We can discover that the Internal Medicine, Surgery and Internal Medicine are the most concerned divisions in the dataset. Therefore more medical efforts should be attached to these divisions in hospitals. While the number of inquiries about Internal Medicine has reached 18327, the amounts of questions about Genetics or Medical Examination are under one hundred. The number of questions over the categories is severely imbalanced.

**Table 3:** The frequency distribution over the categories

Internal Medicine	18327	Cosmetology	775
Surgery	13511	Drugs	529
Gynecology	8691	Health Care	439
Pediatrics	5312	Assistant Inspection	430
Dermatology	4969	Rehabilitation	276
Ophthalmology &	3983	Home Environment	253
Otolaryngology		Child Education	247
Oncology	2118	Nutrition and Health	172
Mental Health	1536	Slimming	169
Chinese Medicine	1452	Genetics	86
Infectious Diseases	1360	Medical Examination	64
Plastic Surgery	1211	Others	31

### Convolutional Semantic Clustered Representation

CNN has been successfully applied to neural language processing in many fields as an advanced feature representation including text classification [26], sentence modeling [27], and QA [28]. It can capture local features using convolving filers [16]. Based on this consideration, we assume that filters in CNN can learn to identify clinical terms and generate their representation.

**Fig. 2:**
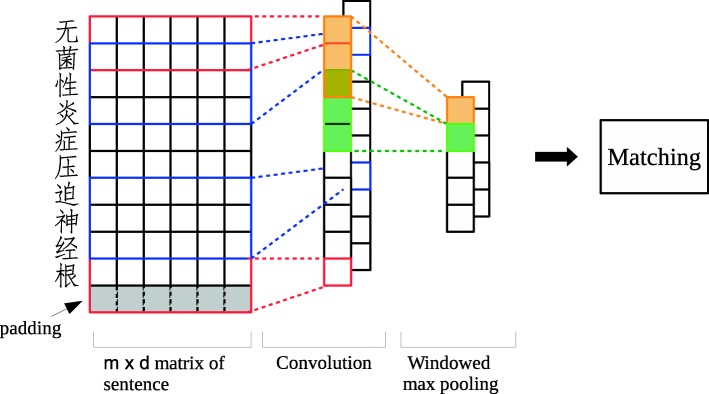
Illustration of CSCR with a character-level input. *m* is the length of input sentence and *d* is the length of embedding for each character

The Convolutional Semantic Clustered Representation (CSCR) model employs CNN to automatically recognize the words and terms by Max pooling around the neighborhood, inspired by the Very Deep Convolutional Neural Networks (VDCNN) [29]. The architecture of CSCR is illustrated in Fig. 2.

Let $x_{i}\in \mathbb {R}^{k}$ be the *k*-dimension character embedding corresponding to the *i*-th character in the sentence. A sentence of length *n* is represented as

1 $$ \mathbf{x_{1:n}}={x_{1}}\oplus {x_{2}}\oplus \ldots\oplus {x_{n}}  $$

where ⊕ is the concatenation operator. For a filter $\mathbf {w} \in \mathbb {R}^{h\times k}$, which is applied to a window height of *h* characters to produce a feature *c*_*i*_, the convolution operation is formulated as

2 $$ c_{i}=f(\mathbf{w}\cdot\mathbf{x}_{i:i+h-1}+b)  $$

where **x**_*i*:*i*+*h*−1_ indicates the concatenation of characters **x**_*i*_,**x**_*i*+1_,…,**x**_*i*+*h*−1_ and *b*∈**R** is a bias term and *f* is a non-linear function such as **tanh** and **ReLU** [30]. This filter is applied to each possible window of characters in the sentence with padding to produce a feature map:

3 $$ \mathbf{c}=[c_{1},c_{2},\ldots,c_{n}]  $$

with $\mathbf {c}\in \mathbb {R}^{n}$. Notice that we get a feature map of the same length of sentence because of padding. We then perform a max-over-time pooling operation with window size *m* for every step with stride length *d* (*d* is a factor of *n*). Practically, we find the max signal among *m*=3 and set *d*=2 to have a convolution result overlapped. Then we get a vector of max values $\hat {\mathbf {c}} \in \mathbb {R}^{\frac {n}{d}}$

4 $$ {}\hat{\mathbf{c}}=[max\{\mathbf{c}_{1:m}\},max\{\mathbf{c}_{1+d:m+d}\},\ldots, max\{\mathbf{c}_{n-d:n-d+m}\}]  $$

The idea is to capture the most important composition patterns of characters to form a word or clinical term in each window *m*. The max value vector $\hat {\mathbf {c}}$ is considered as max correlation degrees between all possible terms in a sentence and filter **w**. In other words, it is a representation of clustered terms in regard to filter **w**. This is the process by which one filter related terms are represented. The model uses multiple filters (with various height) to obtain multiple representation of clustered terms. And we concatenate the vectors as matrix $\mathbf {z}\in \mathbb {R}^{\frac {n}{d}\times |filters|} $ with each row as a semantic representation of characters in a certain block (with ${\frac {n}{d}}$ blocks in total):

5 $$ \mathbf{z}=\left[\hat{\mathbf{c}}_{1},\hat{\mathbf{c}}_{2},\ldots,\hat{\mathbf{c}}_{\#filters}\right]  $$

Given an input matrix of embeddings, unlike the canonical CNN that resulted in a sentence vector, our model produces a matrix with each row being a vector of clustered semantic signals. That means our model enables word-level semantic matching in the following operations.

### Deep Matching Networks

After clustering the characters into latent medical terms and representing a sentence as matrix *z*^′^, we need to compute the matching degrees between the clustered representation of a question-answer pair for identifying whether the answer is the best one. We introduce two different models for semantic matching: multiple positional sentence representation with Long-short Term Memory (MV-LSTM, [22]) and MatchPyramid [23] in this paper. MV-LSTM was a basic matching model that has steady performance. MatchPyramid is the state-of-the-art model for text matching.

#### MV-LSTM

**Positional Sentence Representation** It utilizes a bidirectional LSTM [20] to generate two hidden states to reflect the meaning for the whole sentence from the two directions for each word. The positional sentence representation can be produced by concatenating them directly. Using LSTM, for the forward direction we can obtain a hidden vector $\overrightarrow {h} $ and obtain another $\overleftarrow {h}$ for the reverse direction. The representation for the position *t* in a sentence is $p_{t}=\left [\overrightarrow {h}_{t},\overleftarrow {h}_{t}\right ]^{T}$, where (·)^*T*^ stands for transpose operation for a matrix or vector. For the sentence of length *l*, and dimension size *d* (here *d*=*#fileters*) of each position representation for each word, we finally get a matrix of size *l*×*d* as the semantic representation of the sentence.

**Interaction between Two sentences.** After the representation of the sentence, each position of the question *Q* and answer *A* will interact and compute a similarity score matrix $S\in \mathbb {R}^{m \times n}$ (*m* is length of question matrix *Q* and *n* is the length of answer matrix *A*) using the bilinear matrix $B \in \mathbb {R}^{d\times d}$ (here *d*=*#fileters*). Each element *sim* of matrix *S* is computed as follows:

6 $$ sim\left(\overrightarrow{Q_{i}},\overrightarrow{A_{j}}\right)=\overrightarrow{Q_{i}}B \overrightarrow{A_{j}}+b  $$

where *i,j* denote the *i*^*th*^ and *j*^*th*^ row in *Q* and *A* respectively, *B* is the bilinear matrix to re-weight the interactions between different dimensions in vectors and *b* is the bias. In this way, we can compute a similarity score matrix of size *m*×*n* with each element denoting the score of two corresponding vectors. We do not use the Tensor Layer for faster speed and smaller storage. This also simplifies the model and make its structure more clear.

**Interaction Aggregation** Once we compute the similarity score matrix between two sentences, k-max pooling will be used to extract the most *k* strongest interactions as vector *v* in the matrix [31]. Finally, we use a MLP to aggregate the filtered interaction signals. We utilize two layers of neural networks and generate the final matching score for a binary classifier as follows.

7 $$ (s_{0},s_{1})^{T}=W_{s}\mathrm{f}\left({W_{r}v+b_{r}}\right)+b_{s}  $$

where *s*_0_ and *s*_1_ are the final matching score of the corresponding class, *W*_*r*_,*W*_*s*_ stand for the weights and *b*_*r*_,*b*_*s*_ stand for the corresponding biases. f represents an activation function, which is *tanh* in our setting.

#### MatchPyramid

Unlike MV-LSTM, MatchPyramid directly uses the word embeddings as text representation. In our system, we use the matrix *z*^′^ as text representation considering each row as a word embedding. A matching matrix *S* is computed with each element *sim* being the dot product of word embeddings from question *Q* and answer *A* respectively:

8 $$ sim\left(\overrightarrow{Q_{i}},\overrightarrow{A_{j}}\right)=\overrightarrow{Q_{i}}\cdot \overrightarrow{A_{j}}  $$

Based on this operation, the matching matrix *S* corresponds to a gray image.

**Hierachical Convolution** Then different layers of convolution are performed. Each convolution layer is applied to the result of the previous operation. Square kernels and ReLU activation are adopted. Dynamic pooling strategy is then used afterward, which is a kind of max pooling in a rectangle area. Then the results are reshaped to a vector and fed to a fully connected layer to predict final matching scores *s*_0_ and *s*_1_ for each question-answer pair.

### Model Optimization

Softmax function is utilized to the matching scores of each class for the binary classifier. Then cross entropy is used as the objective function and the whole model learns to minimizing:

9 $$\begin{array}{*{20}l} loss&=-\sum\limits_{i=1}^{N}\left[y^{i}log\left(p_{1}^{i}+\left(1-y^{(i)}log(p_{0}^{(i)}\right)\right)\right], \end{array} $$

10 $$\begin{array}{*{20}l} p_{k}&=\frac{e^{s_{k}}}{e^{s_{0}}+e^{s_{1}}}, k=0,1 \end{array} $$

where *y*^(*i*)^ is the label of the *i*-th training instance. We apply stochastic gradient descent method Adam [32] for parameter update and dropout for regularization [33].

## Results

In this section, we conduct three experiments on our webMedQA dataset. The first experiments investigate the performance of MV-LSTM with different CWS tools. The second experiment compares the performance of two input units and matching models. In the third experiment, we validate whether the proposed CSCR representation can improve the system’s performance.

### Evaluation Metrics

To measure the precision of our models and the ranking of the gold answers, we use the Precision at 1 (P@1) and Mean Average Precision (MAP) as evaluation metrics. Since there is only one positive example in a list, P@1 and MAP can be formalized as follows

11 $$\begin{array}{*{20}l} &P@1=\frac{1}{N}\sum\limits_{i=1}^{N}\delta\left(r\left(s_{1}\left(a_{i}^{+}\right)\right)\right) \end{array} $$

12 $$\begin{array}{*{20}l} &MAP=\frac{1}{N}\sum\limits_{i=1}^{N}\frac{1}{r\left(s_{1}\left(a_{i}^{+}\right)\right)} \end{array} $$

where N is the number of testing ranking lists, $a_{i}^{+}$ is the *i*^*th*^ positive candidate. *r*(·) denotes the rank of a sentence and *δ* is the indicator function. *s*_1_ is the final score of class 1 produced by matching models as in Eq. 7 above.

### Experiment on CWS tools

We use three popular Chinese word segmentation tools including jieba [34], Ansj [35] and Fnlp [36] to split the sentences into tokens and check their influences in the results. We drop all the words that appear in the dataset less than twice. We use MV-LSTM as the matching model here. We set the number of hidden units of bi-LSTM to 100 and the dropout rate is set to 0.5. We set *length*_*q*_=50 and *length*_*a*_=100, since it is the best setting for the MV-LSTM. *k* is set to 50. Word embeddings are randomly initialized with the dimensionality of 200. The hidden size of LSTM is 100. Learning rate is 0.001 and Adam [32] optimizer is used. We use MatchZoo [37] and TensorFlow [38] for implementation. We run the models for 40 epochs and pick up the best performers on the validation set and report their results on the test set. The results are displayed in Table 4 below.

**Table 4:** Performance of different CWS tools on webMedQA with MV-LSTM

	Vocab Size	P@1(%)	MAP(%)
Ansj	44140	57.7	73.5
Fnlp	145058	57.9	74.4
jieba	94630	59.3	75.3

As we can see in Table 4, jieba achieve the highest results in both P@1 and MAP. Ansj performs the worst in these three CWS tools. Considering that Ansj has a smaller vocabulary size, we suppose that the Ansj cuts sentences into smaller segments.

### Experiment on Input Units and Models

In this experiment, we compare the results of using word-based or character-based inputs with BM25, multi-CNN, MV-LSTM and MatchPyramid on our webMedQA dataset.

We use the segmented results from jieba as the word-level inputs since it performs the best. We drop all the words and characters that appear in the dataset less than twice. The vocabulary size for characters is 9648.

For multi-CNN, we set the kernel height to 3 and 4 as in [17]. We use 80 kernels for each size and set the margin to 0.01 for hinge loss. The learning rate is 0.001.

For MV-LSTM, the parameter settings for word-based input are identical to the first experiment above. For character-based input, we set *length*_*q*_=200 and *length*_*a*_=400.

For Matchpyramid, the convolution kernels are of size [3,3] and 64 kernels are used. As for dynamic pooling, the size is set to [3,10]. Other parameters are the same as MV-LSTM. We train these models for 50 epochs. Results are given in Table 5.

**Table 5:** The performance of different matching models using character-level and word-level inputs

Input Unit	Model	P@1(%)	MAP(%)
	Random	20.0	45.7
Char	BM25	26.6	51.2
	multiCNN[17]	39.8	60.1
	MV-LSTM	58.1	74.5
	MatchPyramid	66.0	79.3
Word	BM25	23.6	49.0
	multiCNN[17]	40.0	60.5
	MV-LSTM	59.3	75.3
	MatchPyramid	58.8	74.9

We can see from Table 5 that matching models outperform baselines substantially. It tells that capturing the semantic similarity at the word level enable the model to achieve great improvement.

BM25 performs the worst, only 6.6% higher than random choice in P@1. It shows that the questions and answers in our dataset share very few common words, which make the task difficult. The performance of multi-CNN [17] with word-based and character-based is close and only achieves 40.0% P@1 and 60.1% MAP. The same input unit performs differently when using various matching model. As for MV-LSTM, it achieves 59.3% P@1 and 75.3% MAP with word-based input, 1.2% higher than with character-based input. In contrast, MatchPyramid performs better when using character-based input, with the highest P@1 of 66.0% and MAP of 79.3%. It is 7.2% and 4.4% better than the results of word-based input in P@1 and MAP respectively.

### Experiment on CSCR

**Fig. 3:**
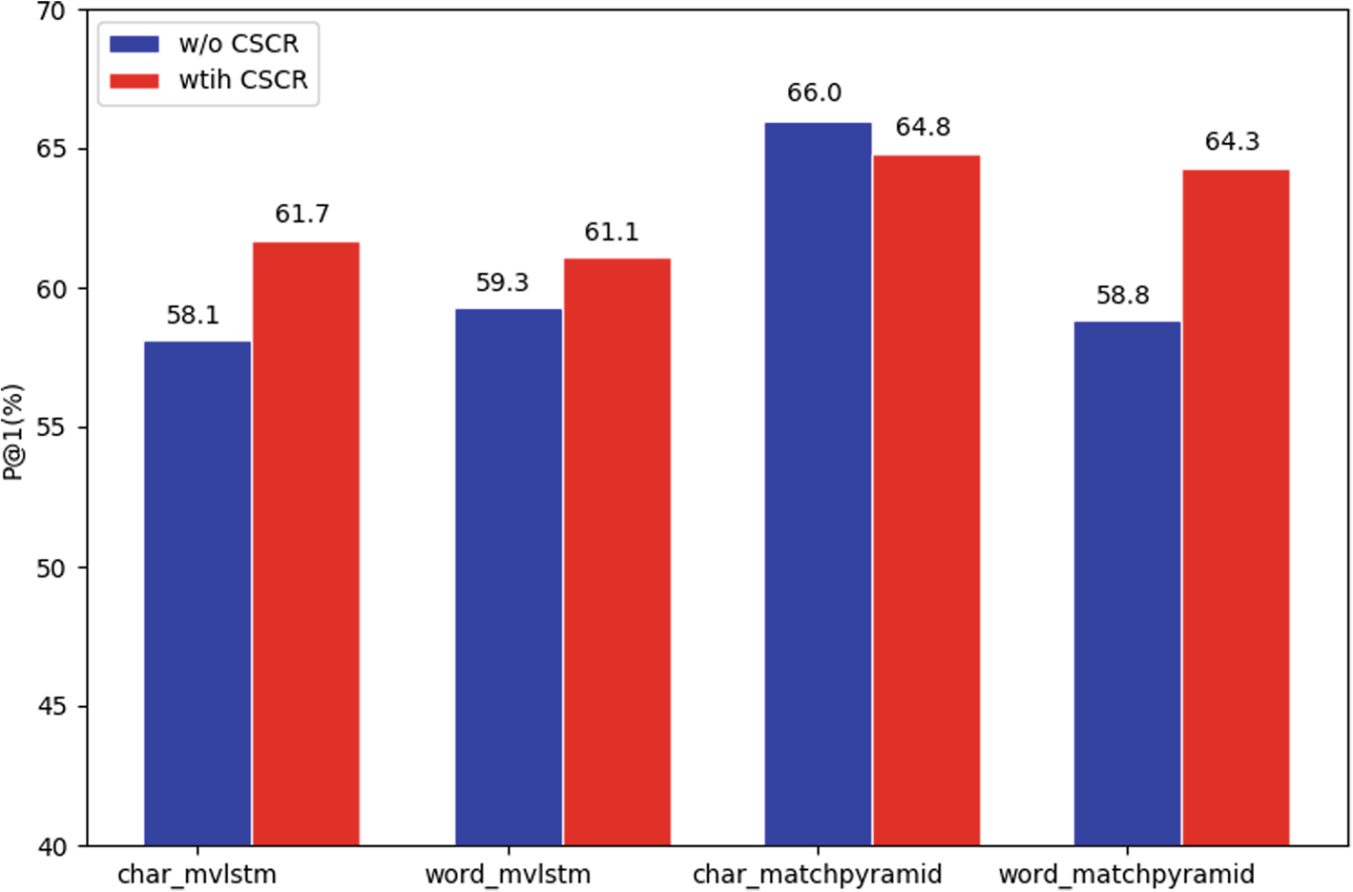
P@1 of matching models with and without CSCR using different input units

**Fig. 4:**
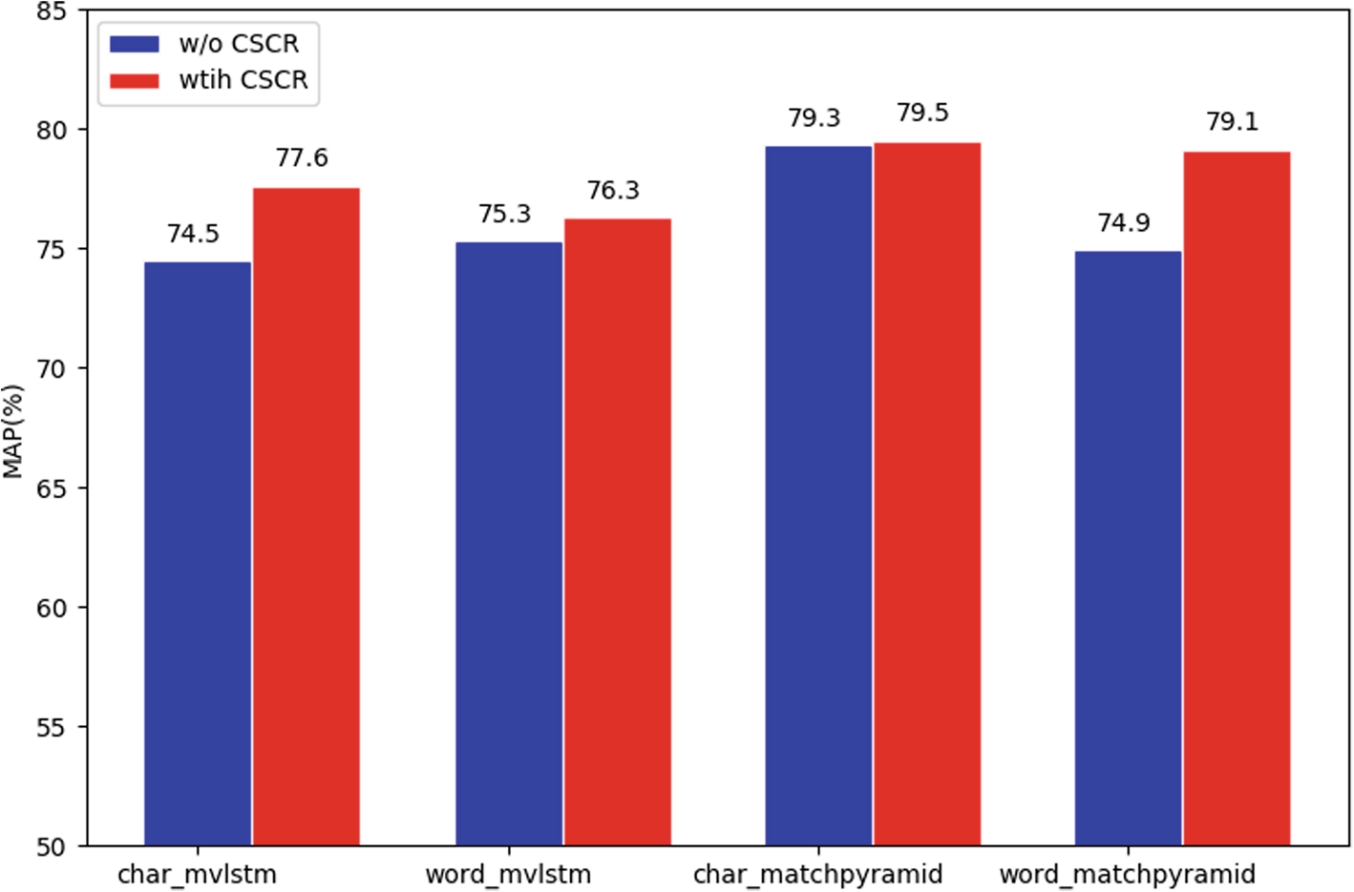
MAP of matching models with and without CSCR using different input units

In this experiment, we validate whether the proposed CSCR model can generate better representation given input of different granularities. We add CSCR to both MV-LSTM and MatchPyramid. For MV-LSTM, the kernel heights are set to [1,2,3] and 64 kernels are used for each size in our experiment. For MatchPyramid, the kernel heights are set to [2,3,4]. Other parameter settings are the same as in the second experiment above. The results are in Fig. 3 and 4.

Figure 3 compares the P@1 results of models with and without CSCR. It is interesting in this figure that CSCR improves the performance of MV-LSTM no matter what input unit it uses. It improves the P@1 of character-based input by 3.0%. Character-level and word-level inputs do not influence the performance of the model with CSCR. Moreover, character-based input with CSCR outperforms word-based input without CSCR. Positive results can also be observed in Fig. 4 for MV-LSTM.

However, for MatchPyramid, the results are complicated. The system with CSCR using word-based input gains 5.5% improvement in p@1. CSCR improves the MAP by 4.2% when using word input. But there is no significant improvement when using characters. Using characters as input directly is the best choice for this model. It can achieve a record of 66.0% in P@1 and 79.3% in MAP, which serves as a competitive benchkmark on webMedQA.

## Discussion

### The most suitable CWS tool for our dataset

**Fig. 5:**
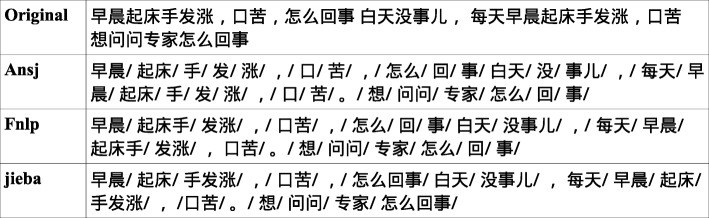
The segmentation results of CWS tools on a sample. Segments are separated by /

Jieba performs best among three CWS tools in the first experiment. Segmentation results produce by Ansj, Fnlp and jieba on the same sample are listed in Fig. 5 below. As we can see, both Ansj and Fnlp produce wrong segmentation results. Ansj cuts words into smaller pieces. e.g., “” and “” are cut into “” and “”. Fnlp regards two words as one word. e.g., “” and “” are merged to “”. In these tools, jieba performs the best on our medical corpus.

### Word-based input v.s. character-based input

Based on our experiments, results of character-based overtake word-based input except for multi-CNN and MV-LSTM without CSCR. It can be attributed to the CWS failure in the medical domain. There is no significant difference between these two input units with multi-CNN, which is opposite to the conclusion from Zhang et al. [17]. It is plausible that we randomly initialize the word or character embeddings instead of using the pre-trained embeddings. Training word vectors based on incorrect word segmentation results may harm the performance and Zhang et al. did not compare the results of word-based and character-based inputs without pre-training the embeddings. MV-LSTM with characters as input performs worse than with words. Based on this phenomenon, we discover that MV-LSTM should use finer inputs since it fails to cluster semantic units based on characters. For MatchPyramid, feeding characters as input perform better. It is plausible that small convolutional kernels and hierarchical CNN layers in MatchPyramid can capture richer details and generate fine-grain representations, which is more suitable for character level inputs than word level inputs.

### Deep matching models outperform multi-CNN

Multi-CNN achieves a worse result on our dataset than on cMedQA dataset. This may attribute to the difficulty of our task. cMedQA data are from one website, therefore, have high consistency while our data are collected from various websites. Moreover, the average lengths of questions and answers in our dataset are shorter (87 v.s. 117 and 147 v.s. 216). Our data are also more conversational. Therefore, our task is more challenging than cMedQA. Deep matching models outperform multi-CNN substantially. It is plausible that MV-LSTM and MatchPyramid learn the relationship between words or sub-words, which is beyond the ability of multi-CNN. Take the sample in Fig. 1 as an example. Matching models can learn the correlation between words in question and answers (e.g., “”/hormone, “”/imbalance, “”/acne in the question and “”/nurse, “”/water, “”/exercises, “”/sleep in the answer) then select the top scores to make a decision. Multi-CNN filters out the important words and produces a representation of these two groups of words respectively. Then the cosine distance of these representations is used as the ranking evidence. But the semantic similarity between these two groups of words is low. Therefore, matching models can capture the word level relationship and have better performance.

### The influence of CSCR

Comparing the P@1 and MAP results of the matching models with different input units, we find that CSCR boosts the performance of matching models in most cases (except the P@1 of MatchPyramid with character-based input). It indicates that CSCR helps the models to achieve better performance by alleviating the negative effect of input units and the CWS problem.

CSCR improves the results of both matching models with word-based input, especially when using MatchPyramid. It is implied that CSCR can produce better representation than CWS results and help to ease the CWS problem in the medical domain.

Character input with CSCR even achieves better results than word input. Therefore, by using the proposed CSCR module, the matching models can achieve better results without CWS than with CWS.

But no increase in character-level input is detected in P@1 when using Matchpyramid. It is partly attributed to the deep CNNs in MatchPyramid. They can capture semantic meanings and extract high-level features from coarse character representations, which makes CSCR unnecessary.

## Conclusion

In this paper, we introduce a large scale Chinese medical QA dataset, webMedQA for research and multiple applications in related fields. We cast the medical QA as an answer selection problem and conduct experiments on it. We compare the performance of different CWS tools. We also evaluate the performance of the two state-of-the-art matching models using character-based and word-based input unit. Experimental results show the necessity of word segmentation when using the MV-LSTM and the superiority of MatchPyramid when using characters as input.

Confronted with the difficulty of word segmentation for medical terms, we propose a novel architecture that can semantically cluster word segments and produce a representation. Experimental results reveal a substantial improvement in both metrics compared with vanilla MV-LSTM with both word and character inputs. But for MatchPyramid, character-based input is the best configuration.

After these experiments, we provide a strong baseline for QA task on the webMedQA dataset. We hope our paper can provide helpful information for research fellows and promote the development in Chinese medical text processing and related fields.

## Abbreviations

CNN: Convolutional neural networks
CSCR: Convolutional semantic clustered representation
CWS: Chinese word segmentation
LSTM: Long-short term memory
MAP: Mean average precision
MLP: Multiple layer perceptron
P@1: Precision at 1
QA: Question answering
